# Self-observation of a virtual body-double engaged in social interaction reduces persecutory thoughts

**DOI:** 10.1038/s41598-021-03373-x

**Published:** 2021-12-14

**Authors:** Geoffrey Gorisse, Gizem Senel, Domna Banakou, Alejandro Beacco, Ramon Oliva, Daniel Freeman, Mel Slater

**Affiliations:** 1 Event Lab, Faculty of Psychology, University of Barcelona, Barcelona, Spain; 2 Institute of Neurosciences of the University of Barcelona, Barcelona, Spain; 3 Department of Psychiatry, University of Oxford, Oxford, UK; 4 Present Address: LAMPA, Arts et Métiers Institute of Technology, Changé, France

**Keywords:** Human behaviour, Information technology

## Abstract

The proportion of the population who experience persecutory thoughts is 10–15%. People then engage in safety-seeking behaviours, typically avoiding social interactions, which prevents disconfirmatory experiences and hence paranoia persists. Here we show that persecutory thoughts can be reduced if prior to engaging in social interaction in VR participants first see their virtual body-double doing so. Thirty non-clinical participants were recruited to take part in a study, where they were embodied in a virtual body that closely resembled themselves, and asked to interact with members of a crowd. In the Random condition (n = 15) they observed their body-double wandering around but not engaging with the crowd. In the Targeted condition the body-double correctly interacted with members of the crowd. The Green Paranoid Thoughts Scale was measured 1 week before and 1 week after the exposure and decreased only for those in the Targeted condition. The results suggest that the observation of the body-double correctly carrying out a social interaction task in VR may lead to anxiety-reducing mental rehearsal for interaction thus overcoming safety behaviours. The results also extend knowledge of the effects of vicarious agency, suggesting that identification with the actions of body-double can influence subsequent psychological state.

## Introduction

Social interaction can make some people feel uncomfortable, especially when it concerns interaction with strangers. Such situations can be even more difficult to manage for people suffering from paranoid ideation. As conceptualized in the paranoia hierarchy^1^, paranoia is a mental disorder manifested by a feeling of persecution that can lead to delusion. Thus, paranoid disorders are characterized in people by their belief that others may have malicious thoughts about them, or even that attempts are being made to harm them. Not limited to patients, the proportion of the population who experience persecutory thoughts is high at around 10–15%^2^. Persecutory ideation relies on several factors including feelings of vulnerability, anxiety and worry^3^. A prominent response and reinforcement of feelings of persecution is to engage in safety behaviours, that is, avoidance of social contact to reduce threat^4^. For example, 96 out or 100 patients studied by Freeman et al.^4^ had engaged in safety behaviours in the month before the study. Simpson et al.^5^ found in a study of 133 students that safety behaviours accounted for 26% of the variance of a measure of paranoia. The use of safety-seeking behaviours prevents the receipt of disconfirmatory evidence, locking the person into the belief system. Safety behaviours are arguably the key maintenance factor in paranoia.

It has been known for a long time that people with a tendency to persecutory delusions in their interaction with others exhibit similar thoughts in relation to interactions with human characters in virtual reality (VR)^6–9^. Extending beyond typical university study samples this result has been found amongst the general population^3^. Hence VR has been used in the study and treatment of paranoia, since it especially affords methods that support the reduction of safety-seeking behaviours through participants essentially practicing social skills in safe environments in conjunction with cognitive-behavioural therapy^10,11^. Here we introduce a new technique where participants first see a double of themselves engaging in social interaction prior to attempting that interaction, thus providing a salient demonstration of overcoming safety behaviours, leading to a reduction in subsequent persecutory thoughts.

Evidence suggests that if you see yourself or even others carrying out a task that you are supposed to do then this encourages you to persevere with that task, or even improve your performance. For example, Fox and Bailenson^12^ found that people who observed virtual copies of themselves performing physical exercises were more likely to engage in physical exercise afterwards than control groups who observed non-self-representations. Fireman et al.^13^ reported that children who observed a video of themselves trying to solve a Tower of Hanoi puzzle were more likely to improve their later performance compared to seeing videos of others doing the same task or doing the task badly.

There are several findings that VR affords out-of-body illusions when a distant virtual body is seen to receive tactile stimulation which is nevertheless felt synchronously on the real body (visuotactile synchrony)^14–16^ or where the distant virtual body moves synchronously with real body movements (visuomotor synchrony), or both visuotactile and visuomotor^17–19^. In particular Lenggenhager et al.^15^ found that participants had the illusion that the body they saw in front was their body and location. When they were moved to a different location and asked to blindly move back to where they started, they moved closer to their virtual body than they had been before, thus providing evidence of an illusory change in location. Galvan Debarba et al.^20^ also showed body ownership over a virtual body seen from third person perspective in VR.

Of particular importance to the current work is the phenomenon of vicarious or illusory agency. Wegner et al.^21^ showed that vicarious agency could be induced by, for example, giving the subject an instruction to move their arms, but where the moving arms seen responding belonged to a confederate standing behind the subject with her arms pushed through under the subject’s armpits whose real arms were out of sight. Banakou and Slater^22^ showed that body ownership over a virtual body that talked also resulted in illusory agency over the talking and also had consequences for later behaviour outside of VR. Body ownership and vicarious agency over the actions of a virtual body that walks even while the participant is seated has also been shown to result in concomitant physiological responses when the virtual body ascends a hill^23^.

Based on this previous research on body ownership and agency over a distant virtual body our aim was to investigate whether observing a body-double correctly carrying out a social interaction task would reduce anxiety in people who have a tendency for paranoid ideation, compared to a control group who saw their body-double carrying out the task incorrectly.

**Figure 1:**
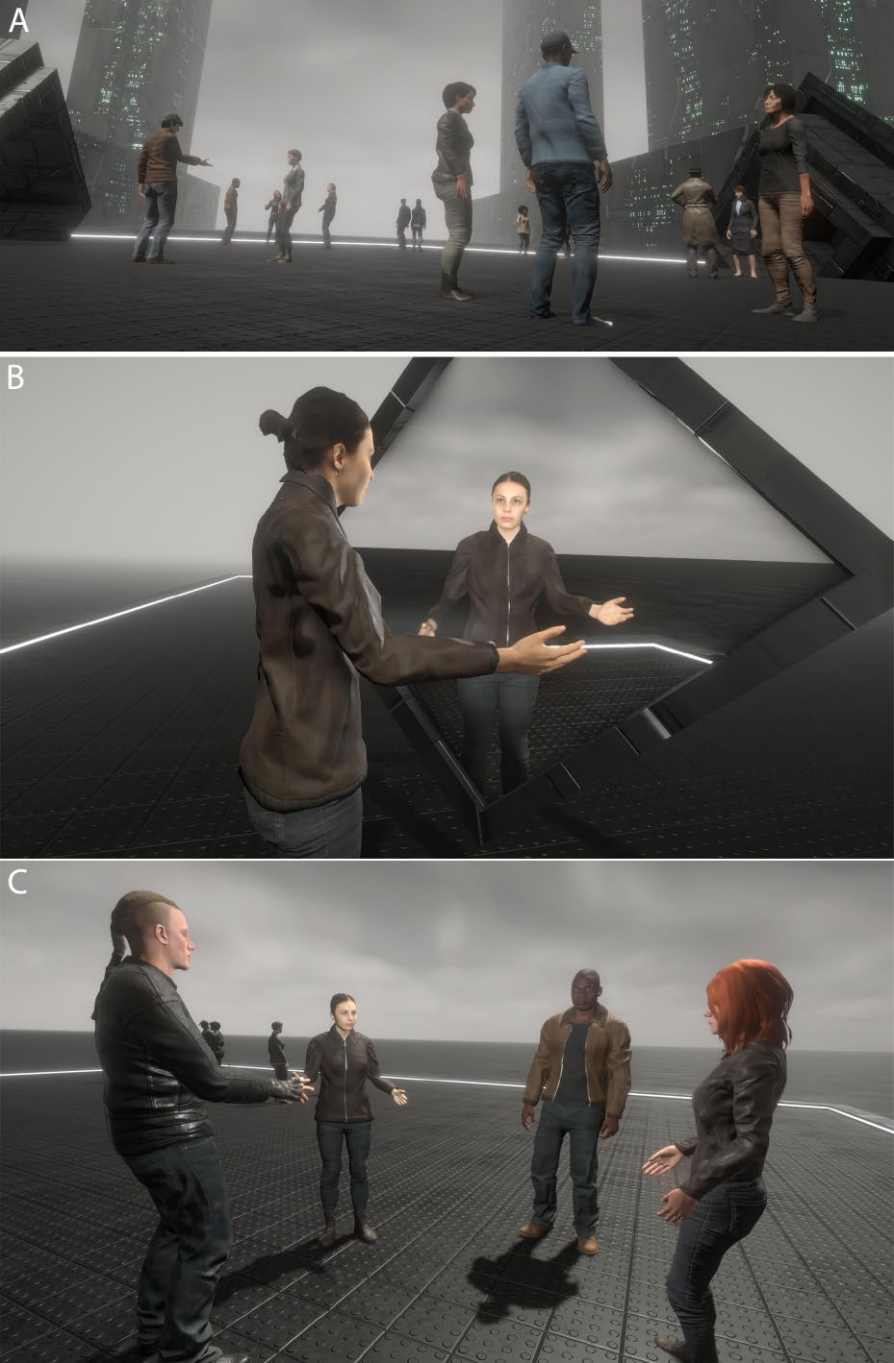
The scenario (**A**) The square between the tall buildings showing the groups. (**B**) A participant sees her body-double in front of the mirror during the embodiment phase before it goes out to the crowd. (**C**) The participant interacting with one of the groups.

Email and advertisements on the University of Barcelona campus were used to recruit the target of 30 participants. During the VR exposure they were asked to approach groups of virtual humans, until they found the group that confirmed that the participant was part of that group, and then listen to the conversation of that group and report back about it. They repeated this three times, and each time a different group responded that they were part of the group. However, before they did this themselves, they saw a virtual body-double emerge from their own embodied viewpoint and carry out the task—either correctly or incorrectly. Hence, participants were randomly divided into two groups of 15 for a between-groups experimental design. In the Random group the virtual double walked out to the environment and wandered around without ever approaching any group, and therefore clearly did not carry out the task. In the Targeted group the virtual double correctly completed the task by approaching each of the groups and engaged in conversation with the last one. After this the virtual double returned and reintegrated into the body of the participant. Then the participant her- or himself actually carried out the approaches to the groups. They repeated this three times, each time being admitted by a different group. Further details are provided in “Materials and methods” section. Figure 1 illustrates the main aspects of the VR exposure, which is also shown in Video S1.

## Results

### Demographics and initial assessment of paranoid thoughts

In the first session participants completed ethics procedures and a demographics questionnaire. The Random and Targeted groups had similar characteristics across a range of demographic measures, shown in SI Table S1. However, in the Random group there were 7/15 males and 3/15 in the Targeted group. The mean ± SD ages are 22.5 ± 4.50 and 24.7 ± 5.43 for Random and Targeted respectively.

The Green et al. Paranoid Thoughts Scale (GPTS)^24^, which was used to assess the tendency to paranoid thoughts, consists of two scales one to assess social reference and the second thoughts of persecution. Changes in the GPTS have been found to correlate with clinical improvement. Social reference refers to (unwarranted) ideas of being the target of the thoughts and actions of others—e.g. “I spent time thinking about friends gossiping about me”. In contrast the questions about persecution revolve around the (unwarranted) idea that others are planning harm to the respondent—e.g. “People wanted me to feel threatened, so they stared at me.” Each scale is composed of 16 items rated on a five-point Likert scale resulting in a range from 16 to 80. Higher scores indicate greater tendency to paranoid ideation.

### Presence, body ownership and agency, and state social paranoia

Participants experienced the VR exposure in the second session 1 week after the first. Immediately following the exposure participants completed the State Social Paranoia Scale (SSPS)^25^ which was developed for assessing paranoia in the context of experimental studies, especially for VR. It has 20 questions, half of them negative statements (‘Someone was hostile towards me’) and the other half more positive (‘I felt very safe in their company’) (with respect to virtual human characters), and the two sets of questions are interspersed. Each question is on a 5-point scale (1 do not agree, to 5 totally agree). Hence the two scales have scores in the range 10 to 50 with higher values meaning more persecutory thoughts (negative) or more neutral or positive thoughts (positive). The two scales are referred to as ssps_neg and ssps_pos. There is no evidence of any immediate social paranoia following the exposure: the ssps_neg scores are low (mean ± S.E. 15.3 ± 2.13 Random, 15.7 ± 2.01 Targeted), and the ssps_pos scores are high (32. 4 ± 1.15 Random, 29.7 ± 1.48 Targeted).

After this they answered a questionnaire regarding presence in the virtual environment, body ownership and agency (Supplementary Table S2). High subjective levels of presence and body ownership and agency are necessary conditions for the experiment to make sense. Therefore, we do not make inferences with respect to these variables to a wider population, we are only interested in the results for this particular sample. The detailed results for these variables are shown in Supplementary Fig. S1. The levels of reported presence, body ownership and agency are high and not different between the Random and Targeted groups, and are similar to previous studies (e.g., Ref.^22^).

### Subject units of distress

Anxiety is tied to paranoia and the type of situation faced by participants could have triggered both. Therefore, at periodic moments during the VR exposure participants responded to a Subjective Unit of Distress Scale (SUDS). This is a self-report tool that allows people to evaluate the anxiety level from 0 (no discomfort at all) to 10 (extremely high discomfort). This was elicited at eight different stages of the experiment (Supplementary Table S3) to assess the evolution of participants’ anxiety levels. This was an easy to administer check for anxiety symptoms that is routinely used in anxiety interventions, and could potentially inform on the most challenging moments, and in an exploratory way to see whether anxiety might decline over time. A validation of the use of SUDS can be found in Ref.^26^. We refer to the scores as *suds1*, *suds2*, …, *suds8*, which mark the SUDS scores at successive time points during the VR exposure. The SUDS were assessed with a pre-recorded voice asking them to report aloud their level of anxiety from 0 to 10.

**Figure 2:**
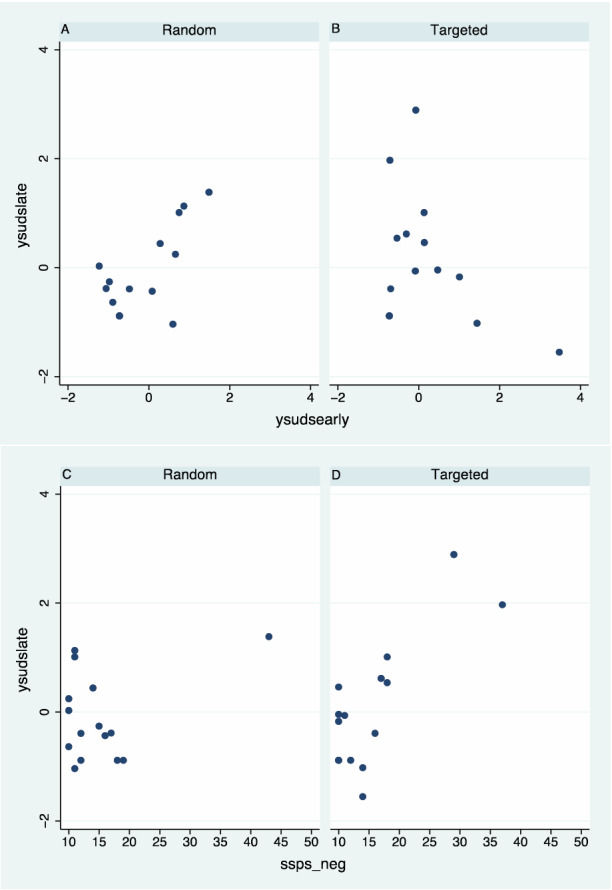
Scatter diagrams for the SUDS and SSPS_neg scores by condition (Random, Targeted). (**A,B**) Factor score ysudslate on ysudsearly. (**C,D**) Factor score ysudslate on SSPS_neg.

In order to analyse the results of the SUDS data we carried out a principal components factor analysis, with varimax rotation. This resulted in two factors, the first accounting for 70% of the variance and the second a further 15%. These factors resulted in two scores, where each score is a linear combination of the SUDS. The scoring coefficients, i.e., the coefficients of each SUDS score in the linear combination indicates a clear break at *suds3*. For the first factor (70%) the coefficients of *suds1* to *suds3* are all negative and the coefficients of *suds4* to *suds8* are positive. Hence this factor is greater for more stress on the later SUDS and we refer to it as *ysudslate*. The second factor (15%) has *suds1* to *suds3* positive and all but *suds5* negative. Hence this factor is dominated by the early SUDS and we refer to it as *ysudsearly*. (Further details of the factor analysis are given in Supplementary Table S4). By construction these two variables are uncorrelated, however, they are not uncorrelated when conditioned on the experimental conditions (Random, Targeted). This is shown in Fig. 2A,B, where for the Random group there is a positive linear relationship between *ysudslate* and *ysudsearly* (Pearson correlation r = 0.69), and a negative linear relationship for the Targeted group (r =  − 0.37). This would mean that the level of stress in the Random group was maintained over time (e.g., if high early on then also high later), but not so for the Targeted group where a high early score is associated with a lower later score.

Since the SSPS questionnaire was administered straight after the VR exposure there should be a relationship between this (in particular SSPS_neg) and the later values of the SUDS (*ysudslate*). These are shown in Fig. 2C,D. For those in the Random condition there is an extreme point which belies interpretation, but for those in the Targeted condition the correlation is clear with r = 0.78. Even combining the two conditions the correlation is high with r = 0.59 (P = 0.0006 as a measure of the strength of the relationship).

### GPTS 1 week later

The GPTS was administered 1 week after the VR exposure to assess the evolution of participants’ paranoia levels. The variable *gpts_ref* (reference) and *gpts_per* (persecution) refer to the two scores, and the suffix ‘*_pre*’ and ‘*_post*’ refer to the scores at the first session and 1 week after the VR exposure respectively.

**Figure 3:**
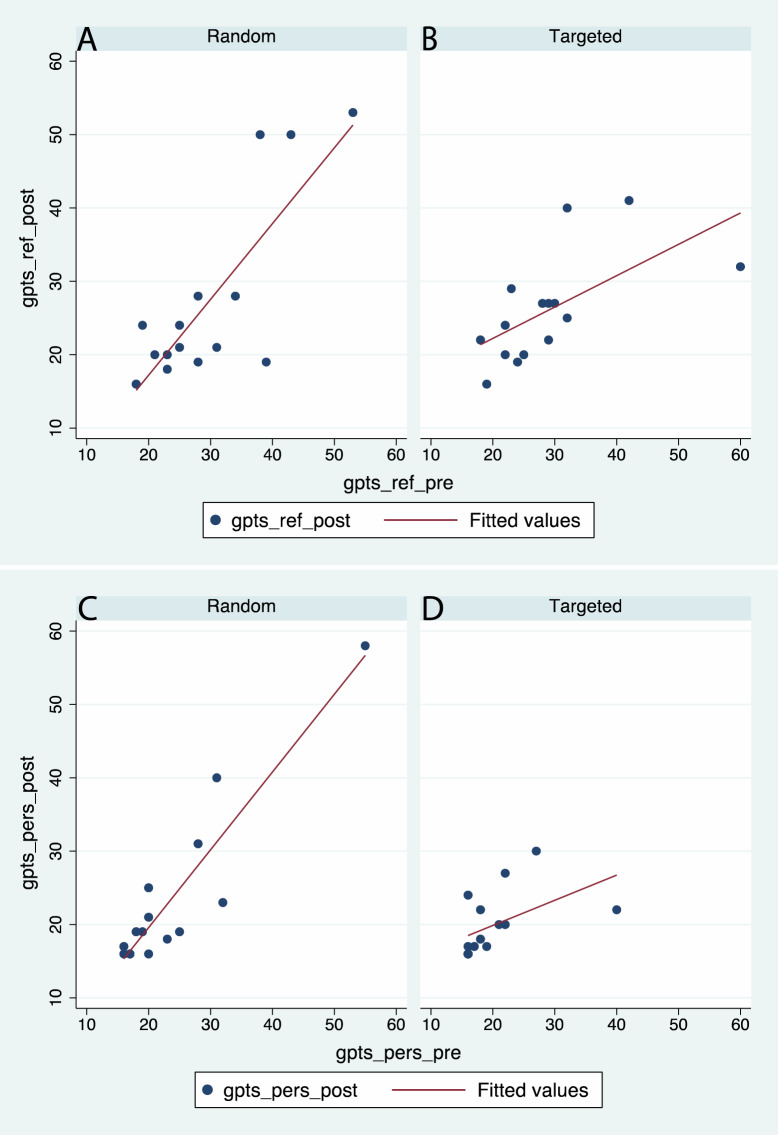
Scatter diagrams and regression lines of the post GPTS scores by the pre GPTS scores, by condition (Random, Targeted). (**A**, **B**) GPTS reference. (**C**, **D**) GPTS persecutory.

Figure 3 shows the scatter plots and corresponding fitted lines of the post scores on the pre scores. Both the social reference scores and persecutory show the same pattern. In the case of the Random condition (Fig. 3A,C) the fitted lines have approximately 45 degree slopes meaning that the post-score reflected the pre-score. However, in the case of the Targeted condition (Fig. 3B,D) the slopes are clearly less than 45 degrees, meaning that the greater the pre-score the proportionately less the post score.

It is important to note that when the slope of the relationship between *pre* and *post* scores is far from 1 then taking the differences *post*–*pre* as the response variable for the experimental condition is not valid. Consider that $post=a+b\times pre$ (approximately). Then $post-pre=a+\left(b-1\right)\times pre$. Only in the case that $b\approx 1$ is it valid to consider the differences. If $b<1$ then greater values of $pre$ lead to smaller values of $post-pre$ irrespective of the effect of the experimental condition. In other words, the size of $pre$ is confounded with the impact of the experimental condition. Therefore we use *pre* as a covariate for the response variable *post*.

### Statistical model

All variable abbreviations are summarised in Table 1. The response variables are: (i) *gpts_ref_post* with *gpts_ref_pre* as a covariate. (ii) *gpts_pers_post* with *gpts_pers_pre* as covariate. (iii) *ysudslate* with *ysudspre* as covariate. SSPS scores are not different between the Random and Targeted conditions and this is not considered further.

**Table 1:** Abbreviations of the variable names and their meanings.

Abbreviation	Meaning
**GPTS**	Green Paranoid Thoughts Scale—to assess the tendency for paranoid thoughts
*gpts_ref*	(Unwarranted) ideas of being the target of the thoughts and actions of others
*gpts_pers*	(Unwarranted) ideas that others are planning harm to the respondent
*_pre*	Measured 1 week before the VR session
*_post*	Measured 1 week after the VR session
**SSPS**	State Social Paranoia Scale
*ssps_neg*	e.g. ‘Someone was hostile towards me’
*ssps_pos*	e.g. ‘I felt very safe in their company’
**SUDS**	Subjective Units of Discomfort
*suds1, …, suds8*	The SUDS scores elicited at 8 successive times during the VR session
*ysudsearly*	This variable is greater the more that there is stress on earlier SUDS scores suds1, suds2, suds3
*ysudslate*	This variable is greater the more that there is stress on later SUDS scores suds4,…,suds8
*identify*	Questionnaire score on “I felt an identification with the virtual double”

There is one binary factor Condition (Random, Targeted). The statistical model is equivalent to a classical analysis of covariance with one covariate and an interaction term. Let ${y}_{ij}$ denote any of the response variables in condition $i$ (1 Random or 2 Targeted), for the $j$ th individual $(j=1, 2, \dots , n=15)$, and let ${\mu }_{ij}$ be the corresponding mean. Then, with ${x}_{ij}$ as a covariate:

1 $${\mu }_{ij}=\mu +{\alpha }_{i}+\beta {x}_{ij}+{\gamma }_{i}{x}_{ij.}$$

$\mu$ is the general mean, ${\alpha }_{i}$ is the main effect of condition $i$ (= 1 for Random, 2 for Targeted), $\beta$ is the main effect of the covariate, and ${\gamma }_{i}$ is the interaction term allowing the slope to be different between the conditions. The constraints on the parameters are as usual for an analysis of variance model:

2 $$\begin{gathered} \alpha_{1} + \alpha_{2} = 0, \hfill \\ \gamma_{1} + \gamma_{2} = 0. \hfill \\ \end{gathered}$$

Of course, the parameters will be different for each of the response variables.

In the case of (i), *gpts_ref_post*, (Eq. (1)) models the linear relationship with *gpts_ref_pre*, but allowing this to be different depending on condition (Random, Targeted). For example, if ${{\gamma }_{1}=\gamma }_{2}=0$, but $\beta \ne 0$ then there is a linear relationship between *gpts_ref_post* and *gpts_ref_pre* which is the same for each condition apart from the intercept $(\mu +{\alpha }_{i})$. If, in these circumstances, ${\alpha }_{2}<0$ then this would mean that there are two theoretical parallel lines, with the one for Targeted lower than the one for Random. If, however, ${\gamma }_{2}<0$ $({\gamma }_{1}>0)$ then the slope of the line of *gpts_ref_post* on *gpts_ref_pre* in the Targeted condition is smaller than the Random condition. This would mean that *gpts_ref_post* is proportionately less in the Targeted condition than the Random condition for every level of *gpts_ref_pre* (Fig. 3A,B). Similar considerations apply for (ii) and (iii).

A Bayesian analysis is used since all of the response variables can be considered simultaneously in one overall model with each equation of the form (Eq. (1)). Hence there is not the problem of loss of significance due to multiple statistical tests since all probability statements are derived from the joint posterior distribution of all parameters. Also, the Bayesian method is flexible, since rather than assume that everything has a normal distribution we use, in this case, a Student t distribution which can incorporate potential outliers. For such reasons, and given doubts about null hypothesis significance testing^27,28^, Bayesian statistical methods have been increasingly employed, including in psychology^29,30^.

From Figs. 2 and 3 it can be seen that there are potential outliers, so a distribution for the ${y}_{ij}$ conditional on the parameters (the likelihood) needs to have a wide support compared to the normal distribution. The likelihood equations for each response variable are therefore of the form:

$${y}_{ij} \sim {{Studen{t}\_{ }}t \left(\upsilon ,{\mu }_{ij},\sigma \right)},$$

where $\upsilon =4$ is the degrees of freedom parameter, ${\mu }_{ij}$ is the mean, and $\sigma$ is the scale parameter. The degrees of freedom $\upsilon =4$ is chosen since this has a much wider effective range than the normal, and further details for this choice are given in Supplementary Text S1. For example, the standard normal distribution (mean 0, standard deviation 1) has 95% of the distribution between ± 1.96. The range for the equivalent Student t distribution with 4 degrees of freedom is ± 2.78.

The prior distributions are all chosen to be weakly informative^31^. Weakly informative priors are proper probability distributions, but with wide variance. All parameters $\mu , {\alpha }_{i},\beta ,{\gamma }_{i}$~ Normal (mean = 0, standard deviation = 10), and therefore the prior 95% credible intervals are − 20 to 20. All parameters $\sigma \sim$ Gamma (shape = 2, rate = 0.1), with the prior 95% credible intervals being 2.5 to 55.7.

The analysis was carried with the Stan programming language^32,33^ (https://mc-stan.org) using the R interface to Stan (https://mc-stan.org/users/interfaces/rstan). The model was run with 4000 iterations and 4 chains, and converged. The factor analysis and the generation of all graphs were carried out with Stata 16.1 (https://stata.com).

### Model results

Table 2 shows summaries of the posterior distributions of the parameters. Note that since ${\alpha }_{2}=-{\alpha }_{1}$ and ${\gamma }_{2}=-{\gamma }_{1}$ only the results for ${\alpha }_{2}$ and ${\gamma }_{2}$ are shown, and these correspond to the Targeted condition. To understand the interpretation of these, consider ${\gamma }_{2}$ in relation to the response variable *gpts_pers_post.* The prior 95% credible interval for this parameter is − 20 to 20, i.e., prior to observing the data we assigned a probability of 0.95 that ${\gamma }_{2}$ is in this wide interval. If after observing the data the posterior distribution assigns the mass of the probability to values ${\gamma }_{2}<0$, this would indicate that greater values of *gpts_pers_pre* are associated with smaller values of *gpts_pers_post*, i.e., that there is a negative association between them. Note that this approach does not involve a test of a hypothesis, but simply updates probabilities in the light of observed data. It can be seen from Table 2 that overall the 95% credible intervals are considerably narrower compared to the prior intervals.

**Table 2:** Summaries of the posterior distributions of the parameters.

Parameter	Mean	SD	2.5%	97.5%	Prob > 0
**gpts_ref_post**					
$\mu$	3.43	4.05	− 4.95	11.04	0.809
${\alpha }_{2}$	6.75	4.00	− 1.48	14.26	0.947
$\beta$	0.78	0.15	0.51	1.09	1.000
${\gamma }_{2}$	− 0.25	0.15	− 0.52	0.06	0.051
$\sigma$	5.26	1.05	3.56	7.60	
**gpts_pers_post**					
$\mu$	3.50	3.25	− 3.49	9.14	0.853
${\alpha }_{2}$	4.85	3.28	− 2.29	10.29	0.916
$\beta$	0.81	0.17	0.52	1.19	1.000
${\gamma }_{2}$	− 0.25	0.18	− 0.53	0.14	0.094
$\sigma$	3.36	0.69	2.21	4.92	
**ysudslate**					
$\mu$	0.02	0.17	− 0.31	0.34	0.552
${\alpha }_{2}$	0.00	0.17	− 0.32	0.34	0.494
$\beta$	0.17	0.17	− 0.17	0.52	0.859
${\gamma }_{2}$	− 0.53	0.17	− 0.85	− 0.19	0.002
$\sigma$	0.70	0.14	0.47	1.01	

For each parameter the table gives the mean, the standard deviation and the 95% credible interval. Prob > 0 is the posterior probability of the parameter being positive.

The three interaction terms have high probabilities of being negative. For GPTS reference the probability $P\left({\gamma }_{2}<0\right)=1-0.051=0.949$, for GPTS persecutory $P\left({\gamma }_{2}<0\right)=0.906$, and for SUDS $P\left({\gamma }_{2}<0\right)=0.998$. These are all posterior probabilities—i.e., conditional on the data. The probability that all three are negative is 0.858 (derived from their joint distribution). Hence for both GPTS scores higher values at Session 1 are associated with proportionately lower values at Session 3 only in the Targeted condition. Similarly greater SUDS scores earlier in the VR exposure are associated with lower SUDS scores later, only in the Targeted condition. These results correspond to Figs. 2 and 3.

Predicted posterior distributions of the response variables can be simulated from the model in order to examine goodness of fit. This results in a probability distribution for each of the three response variables on each individual, i.e., for the different ${y}_{ij}, i=\mathrm{1,2}, j=\mathrm{1,2},\dots ,15$. The means of these distributions give point estimates for each individual on each response variable. The Pearson correlation between these predicted values simulated from the model and the observed values are 0.76 (95% confidence interval 0.56 to 0.88), 0.89 (0.76 to 0.94), and 0.48 (0.15 to 0.72) respectively for GPTS social reference (*gpts_ref_post*), persecution (*gpts_pers_post*) and the SUDS score *ysudslate*. The confidence intervals are not meant as significance tests but only as a way to indicate the strength of the relationships. Hence using the model in (Eq. (1)) to generate new simulated data gives predicted values that are closely related to the true values.

We also carried out a ‘Leave-one-out’ (‘loo’)^34,35^ cross validation method to examine how well the model predicts the data (Supplementary Text S1) which involves predicting the outcome for one data point based on all the remaining data points, for each observation in turn. The analysis shows that the model fits the data well. There is also a detailed explanation of why the degrees of freedom parameter of the Student t distribution was fixed at 4. This method also shows that there is no overfitting.

Justification for the adequacy of the sample size of 30 is given in Supplementary Table S5. We re-ran the analysis using a random sample of 24 (without replacement) from the original data, maintaining an equal number in the Random and Targeted groups (12 Random, 12 Targeted). This results in posterior distributions that are very similar to those of the full sample, and the same conclusions would be reached.

The analysis above concentrates on the impact of the two different approaches of the body-double to the groups (Random or Targeted). The method is premised on participants identifying with the body-double. The model of (Eqs. (1), (2)) can be extended to include the possible impact of different levels of *identify* (SI Table S2). Denote *identify* by $f$, then the model becomes:

3 $${\mu }_{ij}=\mu +{\alpha }_{i}+\beta {x}_{ij}+{\gamma }_{i}{x}_{ij}+\kappa {f}_{ij}+{\lambda }_{i}{f}_{ij},$$

with the constraints on ${\alpha }_{i},{\gamma }_{i}, {\lambda }_{i}$ as in (Eq. (2)). Here $\kappa$ is the main effect of *identify* and ${\lambda }_{i}$ is the interaction.

Fitting this model as before with each of the response variables included leads, of course, to the same results as above for the influence of the Random and Targeted conditions. In the case of social reference (*gpts_ref_post*) the posterior distribution for the interaction term with *identify* ${(\lambda }_{2})$ has 95% credible interval − 1.94 to 1.62, and $P\left({\lambda }_{2}<0\right)=0.578$. For persecutory thoughts (*gpts_pers_post*) the corresponding values are − 2.36 to − 0.31, with probability 0.994 of being negative, and for SUDS (*ysudslate*) − 0.44 to 0.08, with probability 0.931 of being negative. Therefore, there is considerable support for the finding that, in the Targeted condition only, greater identification with the body-double is associated with a decrease in persecutory GPTS in Session 3 compared to Session 1, and support for a decrease in SUDS later in the exposure compared to earlier.

## Discussion

The main finding from this experiment is that observation of a body-double engaging in social interaction (Targeted condition) is associated with a reduction in the GPTS scores 1 week later, relative to initial scores taken 1 week before the exposure (the baseline). When the body-double is seen to walk amongst groups of virtual people but never approaching them (Random condition), then there is no change in the GPTS score relative to the baseline. There is also some evidence that anxiety scores elicited during the exposure (SUDS) are inversely proportional in the later stages compared to the earlier stages in the Targeted condition, but not in the Random condition. There is also a high probability that greater identification with the virtual body further emphasizes the finding for the persecutory GPTS score, but not the social reference score. There is a strong probability that the result for SUDS also is enhanced by greater identification with the body-double for the Targeted group only. The necessary condition of strong average levels of subjective presence, body ownership over the virtual body, and identification over and agency with respect to the actions of the virtual body-double are met, and also there are no differences in these subjective factors between the Random and Targeted groups. So the findings must be due solely to the observation of the body-double correctly carrying out the social interaction.

There is a long tradition of research showing how identification impacts learning. For example Bandura and Huston^36^ found that children mimic incidental behaviour of teachers to the extent that they identified with the teacher, where this was manipulated as the degree of rapport established between student and teacher. Hartley^37^ had groups of children in role play imagine that they were clever, or not clever. Those who imagined they were clever performed better on a cognitive task than the control group. Albright and Malloy^38^ found that when people observed themselves on a video in social interaction with others, they would display better metacognition than a control group who watched a similar video that did not show themselves. Here metacognition refers to each person’s accuracy in predicting the level of anxiety that others in the group would assign to them. Fireman et al.^13^ carried out an experiment where under various conditions children saw videos of themselves or others solving the Tower of Hanoi problem. They found that the best results for improvement in performance were when children observed themselves spontaneously carrying out the task, compared with watching others do the task, whereas attention, success in the task in the video, and emotional valence were not important compared to the spontaneity of the performance.

In the digital realm there has been extensive work on what have been referred to as ‘virtual’ or ‘digital doppelgangers’—that is virtual human characters that look like the participant but which act independently^39^. Bailenson and Segovia^40^ discussed several psychological impacts of participants observing their digital likenesses in virtual reality. For example, participants seeing their virtual doppelganger engage in physical activity are themselves more likely to actually carry out greater exercise during the exposure and demonstrate more physical activity afterwards compared to control groups without the doppelganger^12^. Children exposed to their virtual doppelgangers carrying out various activities tend to develop false memories of themselves having carried those actions^41^. However, Wang et al.^42^ found that the deployment of digital doppelgangers in a teaching task, did not enhance motivation or approach to learning compared to non-look-alike characters. Hatada, et al.^43^ investigated interaction between participants and their digital doppelgangers. The major finding was that participants found their doppelgangers ‘eerie’, especially when they were touched by them, and also because they were unable to control the actions of these look-alike versions of themselves.

There is substantial evidence that when we observe someone doing an action, our brains may simulate doing it. A notable example is an fMRI study when people watched videos of dancers of ballet or capoeira. Corresponding motor cortex activation was found only for those who were expert in the type of dance they watched—indicating that they integrated the actions of others that they were able to comprehend based on their own expertise in the types of motor actions being displayed^44^. Dushanova and Donoghue^45^ observed similar results in specific motor cortex neurons in monkey. Observing actions with which the monkeys were familiar led to neurons firing that would normally be fired when carrying out those actions. This was due either to just observation or mental rehearsal of the action due to the observation.

A critical element in the current experiment is that participants were given an instruction to go out to the square where the groups of people were standing and interact with each group. Wegner et al.^21^ reported vicarious agency when subjects were instructed to move their arms but the arms that moved were those of a confederate, as discussed earlier. In Ref.^22^ participants embodied in a (non-look-alike) virtual body with a reflection in a virtual mirror, saw their virtual body talk, without prior knowledge that this was going to happen. In one group the virtual body moved synchronously with their own movements, and in another group not. The pitch of the voice of the virtual body was always higher than that of the participant. Only participants in the synchronous condition reported illusory agency over the speaking and spoke in a higher pitched voice after the VR exposure. It was argued that for those in the synchronous condition, after the first few words were spoken the CNS inferred agency over the speaking and produced a motor plan to continue speaking in that manner. In order to test whether this result was caused by body ownership or a generalization of the real agency over the virtual body inherent in the synchronous condition, a further experiment induced body ownership through visuotactile stimulation rather than visuomotor^46^. It was found that although illusory agency over the speaking was also reported, there was no effect on the pitch of subsequent speaking. Hence the behavioural result is more likely to be the result of a generalization of real agency to illusory agency than only through body ownership—i.e., via the motor plan.

In the case of the current experiment the instruction to go out to the square and talk to the people was then followed by the virtual double going out to the square and doing that task (correctly or not). The level of illusory agency was high in both conditions (Supplementary Fig. S1C). In the forward model of agency, subsequent to the intention to carry out an act (e.g., after a command) the sensory consequences of the predicted outcome by way of an efference copy are compared with the actual outcome. There is an internal inverse model of how the goal would be achieved, in other words a simulation of the actions necessary to achieve the goal^47^. There is also a requirement for a temporal connection between the intention to carry out the action and the corresponding observed sensory consequences^48^. We propose here that following the command, the internal simulation of carrying out the act of seeing the body-double speaking to the groups of people in the square in the Targeted condition showed a possible instance of how the act could be successfully executed, and supported participants in their preparation for the act. Moreover, with a degree of illusory agency and identification with the virtual double, this preparation would have been enhanced: ‘This is *me* carrying out that act’. By the time they actually did it, their CNS had already simulated it, and they had seen an example of it carried out by a virtual character that clearly represented themselves and over which they had illusory agency. Hence the inverse relationship between the SUDS scores later in the exposure and those earlier (Fig. 3B) for those in the Targeted condition and these SUDS scores correlated with the SSPS scale (Fig. 3D).

What is most interesting in our findings is that the that differences between the Random and Targeted conditions were reflected 1 week later in the GPTS scores (Fig. 3), a questionnaire concerned with social reference or paranoid thoughts in everyday life, even though the action was motor behaviour (going out to the crowd). All participants had carried out the task. The only difference between the Random and Targeted conditions was that those in the latter saw their digital doppelganger carry out the action first. The observation of the virtual double correctly carrying out the action was therefore critical. We propose that during the action observation those in the Targeted condition simulated the action including the affective responses that would be associated with approaching strangers and asking them a somewhat loaded question: “Am I in your group?” Then having already simulated the action and seen it carried out, they carried out the action themselves and could compare their responses during their simulation and during the action observation, with their actual responses. Those in the Random group saw their virtual double carry out the task incorrectly. This would add no positive information about task performance, and for those participants with greater propensity to persecutory thoughts, it would illustrate their normal avoidance behaviours. This would imply that the mental rehearsal of the motor activity and observing the behaviour of their body-double, carried over to affect, and better prepared participants in the Targeted condition to carry out the task, but confirmed avoidance behaviour of those in the Random condition. This difference lasted at least 1 week. Hence in this study we have brought together ownership and agency over a distant virtual body, observation of a body-double carrying out an action and a consequent psychological pay-off in terms of reduced reported persecutory thoughts one week after the experience.

We have proposed an explanation of the results in line with previous findings on vicarious agency, based on the idea that seeing the body double carry out the task invoked mental rehearsal. However, there are other possibilities that cannot be ruled out on the basis of these data. The first is that we do not know if it is necessary for the participant to actually carry out the task. Perhaps only observing the virtual doppelganger would have been sufficient. There are several studies that show that mental rehearsal can lead to improved performance. A meta study by Feltz and Landers^49^ showed this in relation to motor skill, it has been shown for table tennis^50^, and simulation of the process of carrying out a task results in better performance compared to simulating the outcome^51^. Zhang et al.^52^ showed that mental rehearsal of recycling household waste led to more actual recycling behaviour, thus influencing an intention and subsequent action. Crisp and Turner^53^ showed that mental rehearsal of social contact with out-groups can lead to improved intergroup relations. We suggest that mental rehearsal followed by actually carrying out the task would be likely to enhance sensorimotor preparedness and performance even further for motor tasks and facilitate mental preparedness for social tasks. However, we do not have evidence that actually carrying out the task is a necessary part of the method proposed in this paper.

Our findings suggest that our method could be used to help patients overcome their typical avoidance seeking behaviours in real life as well as in VR, although we do not have experimental evidence for this. A meta study by Morina et al.^54^ considered whether virtual reality based psychological therapy resulted in improved outcomes in real life situations. The study supported the findings that behavioural assessments of patients undergoing VR psychological therapy showed improvements compared to before treatment, and in comparison to a waiting group, and that such therapy was at least as effective as face-to-face therapy. In the context of persecutory delusions Freeman et al.^10^ found that VR based therapy led to significant reductions in real-world distress. Hence the evidence does point to the efficacy of VR experience in changing real world outcomes.

The use of VR in psychological therapy has been well-established since the 1990s—for example^55^. A recent meta study by Freeman et al.^56^ pointed out the great promise of VR in this field, with the best success shown in anxiety disorders. Research using VR in the context of paranoia started in the early 2000s^6^, first as a means for investigation of the condition, for example^57,58^, and more recently for treatment^59^. The vast majority of these applications use VR as a way to simulate real world events, but with the advantage of being able to do so in the office of the psychologist. The method of using a digital doppelganger to help patients overcome their anxiety and avoidance behaviours presents a potential further way forward, that goes beyond what is possible in reality.

## Materials and methods

### Participants

Ethics approval was obtained from the Comisión de Bioética de la Universitat de Barcelona (IRB00003099), and participants gave written and informed consent. The experiment was performed in accordance with relevant guidelines and regulations, and in accordance with the Declaration of Helsinki.

Recruitment was carried out by e-mailing the laboratory’s database for participation in experimental studies and through advertisements posted on the campus of the University of Barcelona. Participants were recruited if their GPTS score^24^ was 17 or more on the reference scale. We recruited 20 females and 10 males (30 participants) aged from 18 to 33 (M = 23.6, SD = 5.03). Participants had a correct or corrected vision. Participants were screened through pre-experimental forms for VR contraindications such as alcohol, drug or medication intakes, as well as epilepsy. Amongst those who responded to our advertisements none were excluded. All Participants were native Spanish speakers. They were assigned at random to the two conditions.

On 7-point Likert scales where 1 meant ‘Low’ and 7 ‘High’, they self-reported a median knowledge of information technologies of 4 and a programming skill of 2. All participants had at least one prior virtual reality experience. They remained uninformed about the goal of the study until the last session where they were debriefed.

A total compensation of 20 euros was given for participating in the three sessions, 5 euros after each of the first two sessions and 10 euros after the third.

### Sessions

During the first session, participants were given information about the study to read and sign. They were informed both verbally and in writing that they were free to withdraw from the experiment at any time without giving reasons. After they agreed to continue with the experiment, they were given a consent form to sign and then completed a demographic questionnaire. Then participants were taken to another room with a dedicated lighting setup to scan their upper body to create the 3D model of their virtual double (Fig. 1). After the scanning session, the models were optimized and integrated in the immersive virtual environment allowing participants to embody their virtual double during the second phase of the experiment.

One week after the first session, participants came back to the lab to go through the VR experience. After reading and signing the information sheet of the study and a consent form, they were equipped with the VR devices (HTC Vive pro and Vive trackers) to be immersed in the virtual environment and to be able to control their virtual body.

Once participants were immersed in the virtual environment, they would see their virtual double standing in front of them and facing away. Then, they received the instructions to calibrate their avatar. First, they had to step inside the virtual body in order to see it from a first-person perspective and to press a button of their controller to adjust its size and to enable the movement synchronization. Once the visuomotor synchrony was active, participants were asked to move while looking down at their body and in a mirror alternately in order to enhance the sense of body ownership over their virtual body. Then, they were asked to describe the virtual environment surrounding them and to state their anxiety level on a scale ranging from 0 to 10.

Once the embodiment phase was completed, participants learned to use the navigation metaphors, which combine both teleportation (limited to predefined points) and a natural walking system. This phase required the participants to reach each teleport point and to walk around to collect targets in their surroundings. Then, participants were asked if they fully understood the navigation technique required for the next steps of the experiment. This phase was repeated as many times as necessary.

Once the navigation technique was mastered, participants had to describe the virtual environment which consisted of large skyscraper-style buildings, to report their current level of anxiety, and finally to teleport back to their initial position.

During the next phase of the experiment, participants could observe five groups of virtual humans talking and interacting together (Fig. 1). They were told that their task was to talk with the groups of people that they saw in the square outside. This task consisted in finding the group to which they belonged by approaching and asking them “Am I in your group?”. Each group responded with the answer “Sorry, you’re not part of our group”, in which case the participant moved on to the next group, or “Yes, you’re part of our group” in which case the participant stayed and listened to the group conversation in order to report back about it. This was repeated three times. The first time that they went out the fifth group responded positively, the second time it was the fourth group, and the third time it was the third group.

Prior to participants going out to perform this task, their virtual double emerged from their embodied location and rotated in front of them until it faced them. While it was facing them, the participants’ movements were synchronized with the virtual double. This continued for 20 s. Then, the participants watched as the double turned around and went out to perform the task. Depending on the experimental condition to which the participants were randomly assigned, the virtual double either properly performed the task and spoke the groups of virtual people as required (Targeted condition) or wandered around the space and did not stop and talk with the groups of virtual people (Random condition). For the Random condition the path was programmed and similar for every participant to ensure their body double avoided approaching any group in the scene. Hence ‘Random’ is just a term to label the condition—for it had been truly random then the virtual double might, for example, have walked through some of the groups. When the virtual double returned to the participant’s location and reintegrated into their body, participants reported their current level of anxiety and then they went out to actually do the social interaction task themselves.

They approached each group and asked if they ‘belonged’ to that group. Once participants had found their correct group (the one to which they ‘belonged’), they were invited to listen to the conversation and later report back what they were talking about. This task of going out and finding their group and listening to the conversation was repeated three times, and each time they were accepted by a different group. The content of the dialogues was different for each iteration of the task, they were: Global warming and its consequences on the Earth’s transformation, the effectiveness of the global war on terrorism, and mass supervision systems of society. The conversations had been recorded in advance in Spanish, and played back on the virtual humans, all with appropriate lip sync and body gestures.

When the exposure was finished participants took off the head-mounted display and completed the post-experiment questionnaires to assess their level of paranoia, their senses of presence, co-presence and body ownership during the experiment, as well as their level of agency and identification with the virtual double.

The third and final phase of the experiment took place 1 week after the VR exposure. Participants attended the laboratory to complete the GPTS questionnaire.

### Materials

The head-mounted display used was the HTC Vive Pro. This displayed the 3D virtual environment in stereo with a total resolution of 2880 × 1600 pixels (1440 × 1600 pixels per eye), a field-of-view of 110° and a refresh rate of 90 Hz. We used two Vive controllers and three Vive trackers attached to the participants’ hands, waist, and feet to support real-time full-body tracking using inverse kinematics algorithms. The computer running the application had the Nvidia GeForce RTX 2070 graphics card and an Intel Core i7-8700K @ 3.7Ghz processor.

A Structure Sensor, integrating both a depth and a RGB camera, was used prior to the experiment to scan the participants’ busts. The scanned 3D models were optimized for real-time rendering and integrated on gender and skin matched virtual bodies in the virtual reality application.

The autonomous agents’ voices were recorded using a M-Audio track 2 × 2 interface and a Nova black large-diaphragm condenser microphone. We recorded all the content of the dialogues for each agent in a silent room. All the audio clips have been post-processed to remove artefacts and to equalize the different voices.

### The virtual environment and software

The VR application was developed using the real-time 3D engine Unity (2018.4). The virtual environment consisted of a large square surrounded by buildings and skyscrapers. The central square had five clusters of virtual agents, as well as a few independent agents who wandered around but did not join any of the clusters (Fig. 1). Each cluster was composed of two or three characters who communicated together using both verbal and non-verbal interactions. We developed several behavioural models to make the virtual characters appear plausible and credible to the participants. They were animated using a motion-capture library to ensure that their movements were consistent with their discourse. The probabilistic eye gaze model allowed the autonomous characters to prioritize and focus on important elements of the scene: proximity with other agents or with the participants, talking or moving characters, etc. The lips of the agents were procedurally synchronized with the pre-recorded voices. The content of the dialogues was recorded with as many gender matched volunteers as autonomous agents in the scene.

## Supplementary Information

Supplementary Information 1.

Supplementary Information 2.

Supplementary Video S1.

## Data Availability

Data is available as Supplementary Data S1. Variable names are the same as in the paper. Condition 0 is Random, Condition 1 is Targeted.
