# Supplementary material for: Self-observation of a virtual body-double engaged in social interaction reduces persecutory thoughts
Source: Sci Rep. 2021 Dec 14;11:23923. doi: 10.1038/s41598-021-03373-x (PMC8671390; doi:10.1038/s41598-021-03373-x)
Supplement: Supplementary file 2 — Supplementary Information 2. [file 41598_2021_3373_MOESM2_ESM.docx]

**Supplementary Information**

# Self-observation of a virtual body double engaged in social interaction reduces persecutory thoughts

Geoffrey Gorisse, Gizem Senel, Domna Banakou, Alejandro Beacco, Ramon Oliva, Daniel Freeman, Mel Slater

Mel Slater

Email: [melslater@ub.edu](mailto:melslater@ub.edu)

**This file includes:**

- Supplementary Text S1
- Figure S1
- Tables S1 to S5
- Legends for Video S1
- Supplementary References

# Supplementary Text S1

## Leave-one-out cross validation

We carried out a ‘leave-one-out’ (‘loo’) ^1,2^ cross validation analysis to examine the predictive power of the model. This involves predicting the outcome for one data point based on all the remaining data points, for each observation in turn. The ‘loo’ method estimates a log likelihood criterion *elpd* (expected log pointwise predictive density) and also a parameter to check for overfitting by estimating the expected effective number of parameters (*p_loo*) which should be smaller than the number of observations and also approximate the total number of parameters for the model, as an indication of whether there is model overfitting. The *elpd* is useful for model comparison. The *p_loo* values are 6.8 ± 1.9 (Standard Error), 8.2 ± 3.0, and 5.2 ± 0.8 respectively for GPTS reference, GPTS persecutory and SUDS respectively, indicating no model overfitting.

The loo method also computes ‘pareto k estimates’ for each observation. These provide information about how much the results would change if the corresponding observation were left out – in other words whether points are influential. The theory developed in ^1^ shows that values of $k\leq0.5$ are good estimates, and $0.5<k\leq0.7$ are acceptable, and $k > 0.7$ not. For GPTS reference there is one observation that falls in the acceptable range with $k = 0.53$ and all others have $k<0.5$. For GPTS persecutory all $k < 0.5$ and similarly for SUDS.

The degrees of freedom parameter $\nu=4$ was chosen by choosing the smallest value and the best results for $k$ across all three response variables.

With respect to model fit with the additional parameters to include *identify*, the overall fit is necessarily improved for (Eqn 3) compared to (Eqn 1). However, the improvement is slight, since *elpd* does not increase substantially. For example, in the case of GPTS persecutory the mean and standard error of the increase in *elpd* is 3.6 ± 2.2, the SE being high enough to be compatible with a zero change. Hence although the evidence for identification is strong in the case of GPTS persecutory and SUDS, the more parsimonious model, without including *identify*, is the better one to choose as an overall model, since in terms of model fit there is no difference between the two models.

**Figure S1**. Response to the questionnaires (Table S2). (A) Presence questions (B) Body ownership (C) identification and agency (D) SSPS. The thick horizontal lines are the medians, the boxes are the interquartile ranges (IQR). The Whiskers range from max(min value, lower quartile – 1.5×IQR) to min(max value, upper quartile + 1.5×IQR). Values outside this range are shown individually.

**Table S1**. Demographic information by condition (Random, Targeted)

|  | Random (n=15) | Targeted (n=15) |
| --- | --- | --- |
| Age (mean ± SE) | 22.5 ± 1.16 | 24.7 ± 1.40 |
| Sex (number of females) | 8 | 12 |
| Nationality (number from Spain rather than Latin America) | 12 | 9 |
|  |  |  |
| Education |  |  |
| School | 1 | 1 |
| Graduate | 10 | 9 |
| Postgraduate | 3 | 4 |
| PhD | 0 | 1 |
| Professional | 1 | 0 |
|  |  |  |
| Number of students rather than other work | 13 | 13 |
|  |  |  |
| Information technology knowledge* | 5 (1) | 4 (1) |
| VR experience scale* | 3 (3) | 3 (3) |
| Computer programming knowledge* | 2 (2) | 1 (2) |
|  |  |  |
| Videogames played per year^a^ | 2 (4) | 1 (1) |
| Video games per week^b^ | 0 (2) | 0 (1) |
|  |  |  |
| gpts_ref_pre (mean ± SE) | 29.0 ± 2.55 | 29.9 ± 2.71 |
| gpts_pers_pre (mean ± SE) | 23.8 ± 2.60 | 20.0 ± 1.65 |

*Likert scale 1-7 where 1 is Low and 7 is High. The values shown are the median (IQR).

^a^ 0 = 0, 1 = 1-5, 2 = 6-10, 3 = 11-15, 4 = 16-20, 5 = 21-25, 6 = >25

^b^ 0 = 0, 1 = 1, 2 = 2-3, 3 = 3-5, 4 = 5-7, 5 = 7-9, 6 = >9

**Table S2**. Presence, body ownership and identification questionnaire. Items range from -3 (totally disagree) to +3 (totally agree). The first two questions assess presence, the next three body ownership and the last two identification and agency.

| **Variable** | **Question** |
| --- | --- |
| copres | I had the sensation to share the environment with other people as if I were really with them in the same place. |
| there | I had the sensation to be in the environment (+3 is the sensation that I would normally have in a place). |
| down | I felt that the virtual body that I saw when I looked down was my body. |
| mirror | I felt that the virtual body I saw in the mirror was my body. |
| notme | I felt that the virtual body was not me. |
| identify | I felt an identification with the virtual double. |
| agency | I had the impression that the actions of the virtual double could be my actions. |

**Table S3**. The timing of the SUDS assessments.

| Name | Assessed … |
| --- | --- |
| *suds1* | After the calibration of the virtual body and the embodiment phase. |
| *suds2* | After the explanation of the task before the double appears. |
| *suds3* | After the observation of the double walking outside to the groups and just before they have to go out to find their first group. |
| *suds4* | After they reported the content of the discussion with their first group. |
| *suds5* | Before they have to find their second group. |
| *suds6* | After they reported the content of the discussion with their second group. |
| *suds7* | Before they have to find their third group. |
| *suds8* | After they reported the content of the discussion with their third group, just before the end of the experiment. |

**Table S4**. Principal components factor analysis with varimax rotation of the SUDS (*suds1*,…,*suds8*) of Table S3. Scoring coefficients (method of regression).

| **Variable** | **Factor1** | **Factor2** |
| --- | --- | --- |
| *suds1* | -0.135 | 0.381 |
| *suds2* | -0.222 | 0.477 |
| *suds3* | -0.050 | 0.307 |
| *suds4* | 0.288 | -0.109 |
| *suds5* | 0.110 | 0.130 |
| *suds6* | 0.217 | -0.004 |
| *suds7* | 0.291 | -0.112 |
| *suds8* | 0.353 | -0.231 |

**Table S5**. A random sample of 24 (without replacement) was chosen from the original 30 observations maintaining 12 in each of the Random and Targeted conditions. The 95% credible intervals for n=24 and n=30 are shown (these are the results from Table 2 in the case of n=30).

| **Parameters** | **n = 24** | | | **n = 30** | | |
| --- | --- | --- | --- | --- | --- | --- |
| **gpts_ref_post** | **2.5%** | **97.5%** | **Prob>0** | **2.5%** | **97.5%** | **Prob>0** |
| $\mu$ | -6.12 | 11.38 | 0.760 | -4.95 | 11.04 | 0.809 |
| $\alpha_{2}$ | -1.69 | 15.29 | 0.948 | -1.48 | 14.26 | 0.947 |
| $\beta$ | 0.49 | 1.11 | 1.000 | 0.51 | 1.09 | 1.000 |
| $\gamma_{2}$ | -0.57 | 0.04 | 0.038 | -0.52 | 0.06 | 0.051 |
| $\sigma$ | 3.65 | 8.88 |  | 3.56 | 7.60 |  |
| **gpts_pers_post** |  |  |  |  |  |  |
| $\mu$ | -1.61 | 10.59 | 0.938 | -3.49 | 9.14 | 0.853 |
| $\alpha_{2}$ | -0.84 | 11.29 | 0.958 | -2.29 | 10.29 | 0.916 |
| $\beta$ | 0.46 | 1.08 | 1.000 | 0.52 | 1.19 | 1.000 |
| $\gamma_{2}$ | -0.57 | 0.04 | 0.038 | -0.53 | 0.14 | 0.094 |
| $\sigma$ | 2.28 | 5.72 |  | 2.21 | 4.92 |  |
| **ysudslate** |  |  |  |  |  |  |
| $\mu$ | -0.40 | 0.43 | 0.489 | -0.31 | 0.34 | 0.552 |
| $\alpha_{2}$ | -0.40 | 0.46 | 0.508 | -0.32 | 0.34 | 0.494 |
| $\beta$ | -0.29 | 0.87 | 0.824 | -0.17 | 0.52 | 0.859 |
| $\gamma_{2}$ | -0.87 | 0.32 | 0.140 | -0.85 | -0.19 | 0.002 |
| $\sigma$ | 0.53 | 1.26 |  | 0.47 | 1.01 |  |

# Movie S1 (separate file).

Video illustrating the main features of the experiment.

# Supplementary References

1 Vehtari, A., Gelman, A. & Gabry, J. Practical Bayesian model evaluation using leave-one-out cross-validation and WAIC. *Statistics and computing* **27**, 1413-1432 (2017).

2 Paananen, T., Piironen, J., Buerkner, P.-C. & Vehtari, A. Implicitly Adaptive Importance Sampling. . *arXiv* **arXiv preprint arXiv:1906.08850.** (2020).
